# Cortical and subcortical activities during food rewards versus social interaction in rats

**DOI:** 10.1038/s41598-025-87880-1

**Published:** 2025-02-05

**Authors:** Florbela Rocha-Almeida, Ana R. Conde-Moro, Antonio Fernández-Ruiz, José M. Delgado-García, Agnès Gruart

**Affiliations:** 1 Division of Neurosciences, Pablo de Olavide University, 41013 Seville, Spain; 2 Department of Neurobiology and Behavior, Cornell University, Ithaca, NY 14853 USA

**Keywords:** Rats, Reward preference, Food, Social interaction, Electrophysiology, Theta–Gamma coupling

## Abstract

Balancing food foraging with social interaction is crucial for survival and reproduction in many species of mammals. We wanted to investigate the reward preferences in adult male rats by allowing them to lever-press for both food and social rewards (interaction with another rat), while their performance and electrophysiological activities were recorded. Local field potentials (LFPs) were analyzed across five neuroanatomical regions involved in reward processing, decision-making, and social behavior. Despite ad libitum food availability, rats consistently prioritized food. LFP analysis revealed a decrease in nucleus accumbens (NAc) spectral power following social interaction, accompanied by specific alterations in delta and theta bands within the medial prefrontal cortex (mPFC). The spectral power of LFPs delta and/or theta bands were different for the five selected regions following food reward vs. social interactions. Cross-frequency coupling analysis provided further insights, demonstrating dynamic changes in theta-to-gamma coupling during both food and social rewards, with distinct roles for slow- and fast-gamma frequencies. These findings shed light on the intricate neural processes underlying reward preferences and/or decision-making choices, highlighting the NAc’s potential role in social reward processing, and the mPFC’s involvement in modulating theta–gamma rhythms during reward-related decision-making.

**Supplementary Information:**

The online version contains supplementary material available at 10.1038/s41598-025-87880-1.

## Introduction

In behavioral neuroscience, researchers have long been interested in understanding the motivations that drive animal behavior. Preference tests, where animals are presented with different options and allowed to choose between them, have been widely used to assess motivation^1^. Recent studies have introduced new methodologies for simultaneously presenting two different rewards to animals for direct comparison^2,3^.

In this study, to evaluate rat preferences, we selected two types of rewards: food and social interaction. Food, as a primary physiological need, has long been recognized as a potent reinforcer. Similarly, social interaction has been shown to serve as a powerful reinforcer, influencing behavior in a way comparable to other well-established rewards^4–7^.

**Fig. 1:**
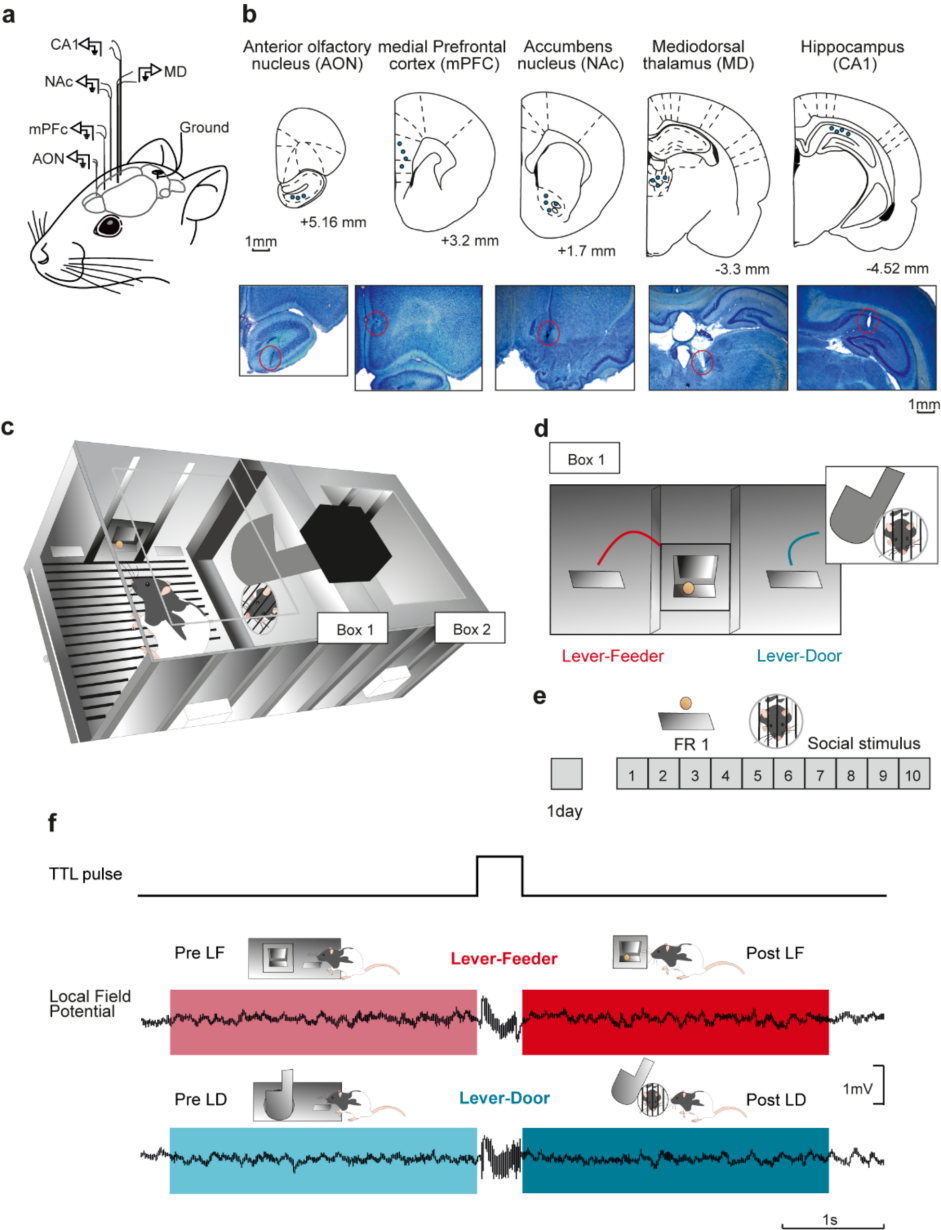
Experimental design. (**a**) Rats (n = 10) were chronically implanted with bipolar tungsten electrodes in the following brain areas: the anterior olfactory nucleus (AON), the medial prefrontal cortex (mPFC), the nucleus accumbens (NAc), the mediodorsal thalamic nucleus (MD), and the hippocampal CA1 area (CA1), all of them located in the right hemisphere. (**b**) Schematic representation of the approximate location of the recording electrodes in each selected brain area (blue dots) and representative photomicrographs illustrating electrode traces. (**c**, **d**) Experimental recording setup. Two modified adjacent Skinner boxes were separated by a guillotine door into two equal-size compartments (**c**). Box 1 contained two available levers: the left lever (Lever-Feeder, LF) provided access to food pellets, with a 1:1 fixed-ratio schedule, while the right lever (Lever-Door, LD) allowed 10 s of visual and partial physical contact with another male rat located in Box 2 (**d**). (**e**) Following two habituation sessions including a wired connection to the recording system, experimental sessions (20 min each) were maintained for 10 successive days. Food rewards were maintained at a fixed ratio of 1:1 (FR1) throughout all the experimental sessions. (**f**) An illustration of a TTL pulse aligned with the activation of lever pressings and LFP samples (3 s each) selected for further representation and analysis. The color code runs as follows: before pressing Lever-Feeder (Pre LF) in light red; before pressing Lever-Door (Pre LF) in light blue; after pressing Lever-Feeder and obtaining the food pellet (Post LF) in dark red; and after pressing Lever-Door and opening the door for social interaction (Post LD) in dark blue.

Two modified and adjacent Skinner boxes were used, one with two levers where the experimental animal (a young adult male rat) was placed (Box 1), and the other box, without levers, containing another male rat as the putative social stimulus (Box 2) (Fig. 1). The use of Skinner boxes is a well-established method for the study of rodent individual and social behaviors and their elective preferences. The versatility and precision in observing rodent behavioral performance, plus the ability to study without interference, make it an excellent choice.

In this paper, we study the selective preferences of rats to choose between food and social interactions while recording local field potentials (LFPs) in five different brain areas: the anterior olfactory nucleus (AON), the mPFC, the NAc, the mediodorsal thalamic nucleus (MD), and the hippocampal CA1 area (CA1). These cortical structures such as the mPFC play important roles in cognitive processes involved in the proper selection of particular behaviors, as well as in their timing and execution^8–16^, and cooperative behaviors^17^, while subcortical structures such as the NAc have been convincingly related to rewarding learning tasks^18–22^, and the MD is an important source of selected sensory information to the mPFC^14^. In addition, the hippocampus is involved in the representations of space and time, but also in the formation of specific memories as well as in their initial storage^23–28^. Finally, the AON plays important roles in spatiotemporal navigation, social recognition processes^29,30^.

By investigating LFP oscillations in these brain areas we expected to obtain specific information on the involvement of neuronal populations in decision-making tasks and potentially link them to ongoing behaviors, correlating them with each selected task. Specifically, we wanted to determine the spectral powers of recorded LFP oscillations and see how they interact across the five selected neural structures. For this, we carried out a cross-frequency spectral analysis—specifically, phase-amplitude coupling—and investigated how the phase of slower oscillations interacts with the amplitude of faster rhythms^31,32^. Specifically, we aimed to investigate how mPFC oscillations influence both directly connected brain areas, such as the NAc and MD^33,34^, and indirectly connected regions, the AON and CA1^35,36^, given the mPFC’s critical role in higher-order cognitive processes, including decision-making and goal-directed behavior.

In short, the main goal of this study was to analyze the spectral properties of neural oscillations recorded from these different brain areas and how they interact with each other to support the decision-making and reward processing, both before the animals made their decisions and when they received the corresponding reward (either a food pellet or a brief social interaction with another conspecific).

Our study shows that while rats consistently preferred food over social interaction, distinct neuronal patterns emerged depending on the reward type. Power of low-frequency bands in areas such as the mPFC, NAc, and CA1 varied, with mPFC modulation differing between rewards: fast-gamma oscillations (80–110 Hz) were prominent during social interactions, and slow-gamma oscillations (30–60 Hz) were observed for food rewards. These findings underline the involvement of specific brain regions in reward processing, suggesting that the consummation of food and social rewards is processed differently at the neuronal level.

## Results

### Experimental design

As depicted in Fig. 1a and b, we implanted chronic recording electrodes in five distinct brain regions of a rat placed in Box 1 within an instrumental conditioning setup. This setup (illustrated in Fig. 1c) consisted of two adjacent, modified Skinner boxes separated by a stainless-steel grid and connected by an intermediate guillotine door. Box 1 was equipped with two response levers, while Box 2 contained no levers and housed an unoperated conspecific. In Box 1, pressing lever 1 delivered a food pellet on a fixed-ratio 1:1 schedule (Lever-Feeder), whereas pressing lever 2 activated the guillotine door mechanism (Fig. 1d), granting limited access to Box 2 (Lever-Door). The door remained open for 10 s, allowing brief social interaction between the two rats.

**Fig. 2:**
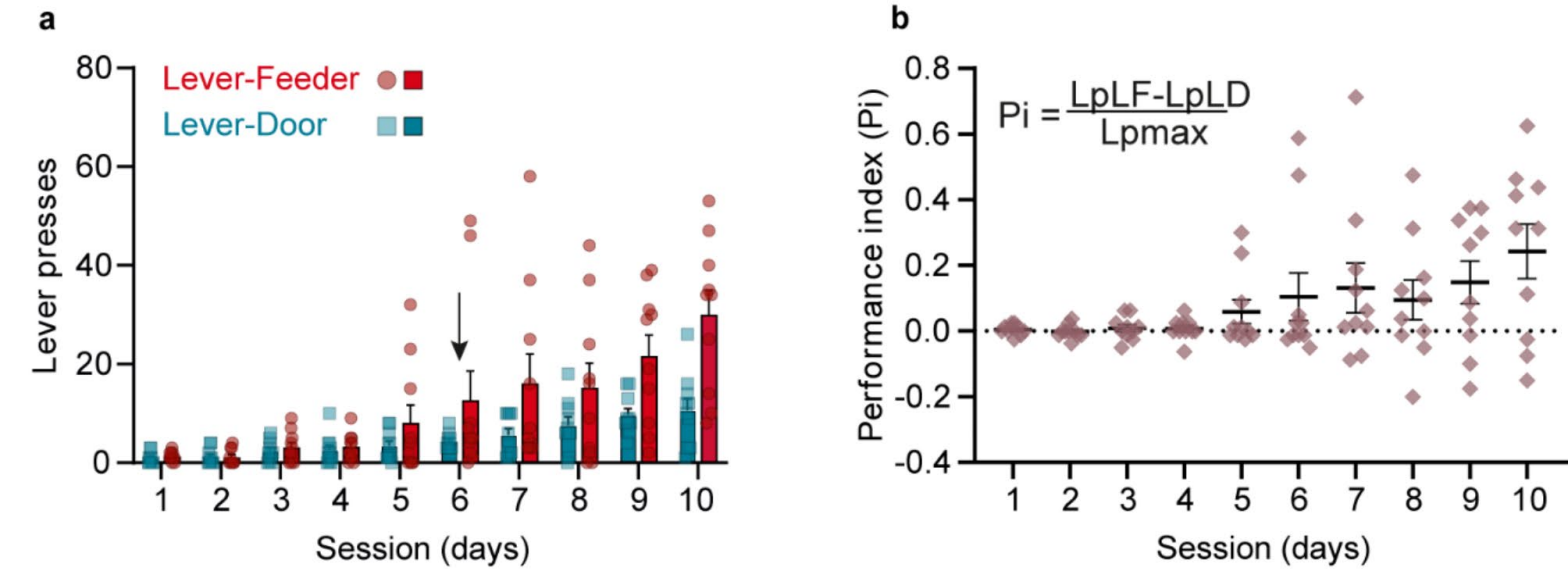
Evolution of rat’s performances across session days. (**a**) A representation of the average amount of lever presses across the 10 recording sessions for Lever-Feeder (red) and Lever-Door (blue) (n = 10 rats). The experimental animal had to choose between Lever-Feeder or Lever-Door, and each time one of these levers was pressed, the animal had to wait 15 s for both to be available again. Red bars illustrate mean ± s.e.m. of lever presses for Lever-Feeder, while dark blue bars illustrate mean ± s.e.m. of lever presses for Lever-Door for each recording session. The arrow represents the day criterion was reached —namely, every rat started to go directly to the expected reward after pressing the corresponding lever. (**b**) A performance index (Pi), defined as Pi = (LpLF-LpLD)/Lpmax); where LpLF = number of lever presses of Lever-Feeder, LpLD = number of lever presses of Lever-Door, and Lpmax = the maximum possible number of lever presses. Lpmax was = 80, because each testing session had a duration of 1200 s and the frequency of availability of levers was 15 s (when the animal pressed a lever, both levers remained retracted for 15 s). 1: Lever-Feeder > Lever-Door; 0: Lever-Feeder = Lever-Door; -1: Lever-Feeder < Lever-Door.

The inclusion criteria for determining whether an animal comprehended the function of each lever were as follows: if the Lever-Feeder was pressed, the animal moved to the feeder to receive a pellet of food; and when the Lever-Door was pressed, the animal moved to the door to interact with the accompanying rat located in Box 2. In short, after pressing the Lever-Feeder, rats would pick the food pellet and briefly chew it near the feeder. Correspondingly, after pressing the Lever-Door, rats would move to the guillotine door and engage in sniffing and more physical interactions with the accompanying rat. A representation of the time spent by the experimental rat in Box 1 and the social stimulus rat in Box 2, is shown in Supplementary Fig. S1. For the quantitative analysis, we selected 10 rats which reached the selected criterion (see Methods) by the 6th recording day (Fig. 2a).

### Comparable lever presses for food and social interactions in adult male rats

From the 6th day onward, there was a tendency for lever presses for food and social interactions to increase over days, although no statistical differences were observed for any of the recording sessions (Fig. 2a). Nevertheless, by day 10, only 3 out of 10 animals had a preference for pressing the Lever-Door rather than the Lever-Feeder (Fig. 2b).

In accordance with the experimental design, the maximum number of lever presses in a session was 80, since the animal had to wait 15 s for the levers to be available again, and the session had a duration of 20 min. On day 10, i.e., the last recording day, the 10 selected animals pressed the Lever-Feeder a maximum of 300 times, corresponding to the maximum number of presses across all days for that lever. For Lever-Door, the total number was 106, also corresponding to the maximum on the same day. The Performance Index (Pi), defined as Pi = (LpLD – LpLF) / Lpmax, where LpLF represents the number of presses on the Lever-Feeder and LpLD represents the number of presses on the Lever-Door (as illustrated in Fig. 2b), further confirmed that, on average, rats preferred to interact with the Lever-Feeder rather than with the Lever-Door.

### Decrease in the spectral power of LFPs recorded in the NAc and the CA1 following Lever-Door press compared to pre-press activity

**Fig. 3:**
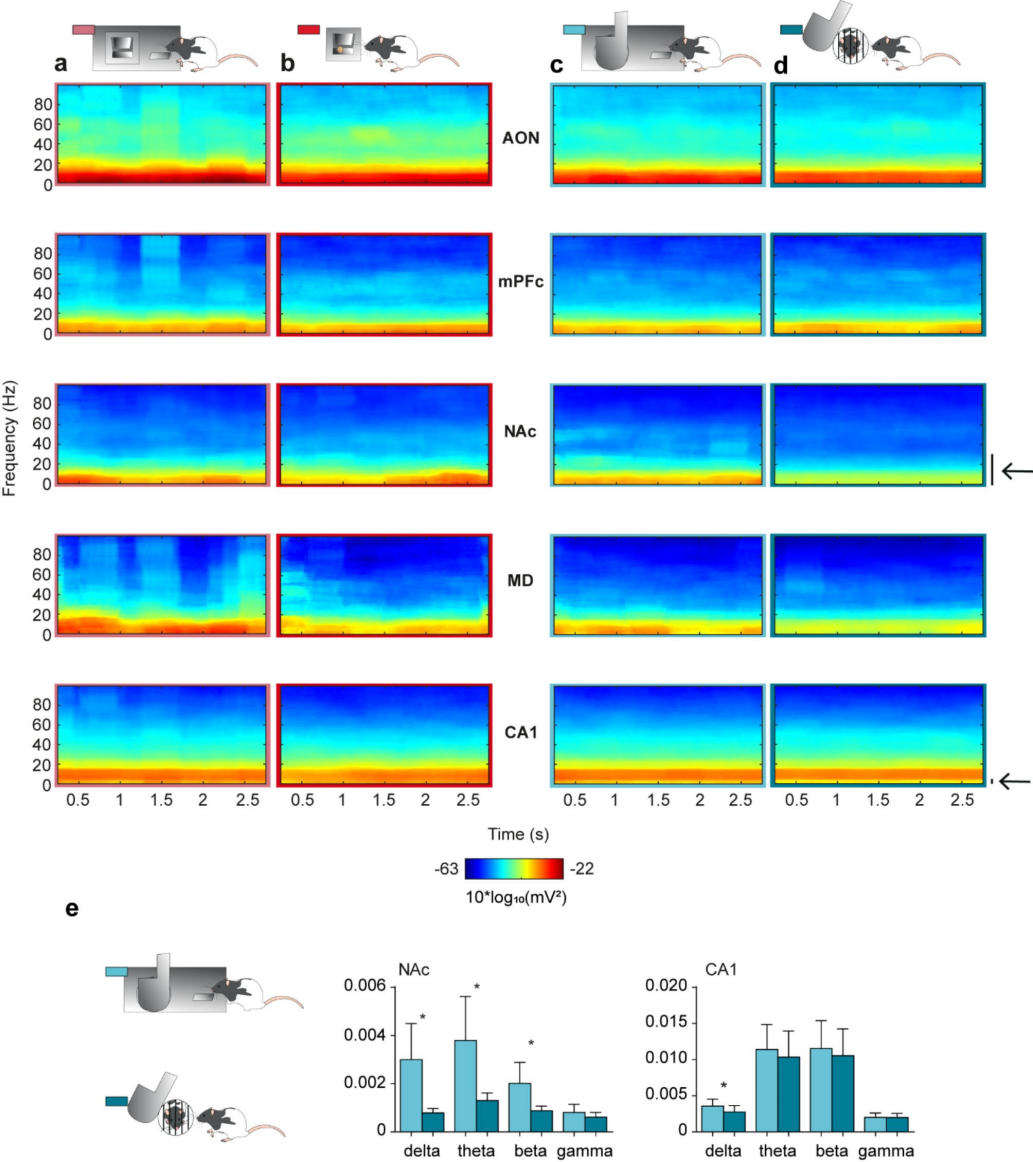
Time–frequency representations of LFPs recorded in the five selected areas (AON, mPFC, NAc, MD, and CA1) during the following experimental situations: (**a**) Pre Lever-Feeder, (**b)** Post Lever-Feeder, (**c**) Pre Lever-Door, and (**d**) Post Lever-Door. Significant differences are marked with black arrows. For the sake of homogeneity, all the selected epochs were 3 s in duration. (**e**) Representation of the quantification of spectral powers from the time–frequency analysis of LFPs of Pre and Post Lever-Door (light blue and dark blue, respectively) that presented significant differences, indicating a selective involvement of some of the recorded areas in social interactions. NAc: delta band, *p* = 0.02; theta band, *p* = 0.02; and beta band, *p* = 0.04. CA1: delta band, *p* = 0.03. Bars represent mean ± s.e.m. Note the evident decrease in spectral power (mostly in delta and theta bands) in some recording areas (NAc, MD) during social interactions as compared with those illustrated during food rewards. The number of samples for each reward (during Pre and Post), animal, and session (from day 6, inclusive) was analyzed uniformly at random by using *randsample* from MATLAB. AON (n = 60 samples; 5 rats); mPFC (n = 57 samples; 6 rats); NAc (n = 73 samples; 7 rats); MD (n = 37 samples; 4 rats); CA1 (n = 80 samples; 7 rats).

In the present experiments, we simultaneously recorded activity from five brain areas: AON, mPFC, NAc, MD, and CA1. Figure 3 shows time–frequency representations of LFPs recorded in these brain areas during 3-s epochs collected before pressing the levers for food (pre-LF) or door opening (pre-LD) and after pressing them, i.e., when the animal could collect the corresponding reward (post-LF and post-LD, respectively). The analysis illustrated in this figure indicated a decrease in the spectral power of delta, theta, and beta bands of LFPs recorded from the NAc after pressing the lever for social interaction [Fig. 3d and e; delta and theta: W_(7)_ = − 28, p = 0.02; beta: W_(7)_ = − 28, p = 0.04, Wilcoxon matched-pairs signed-rank tests, two-tailed]. There was also a significant decrease in delta band power in CA1 in the post-LD situation [Fig. 3e; W_(7)_ =  − 21, p = 0.03, Wilcoxon matched-pairs signed-rank test, two-tailed]. A non-significant decrease was observed in the spectral power of the theta band (7 − 9 Hz) in CA1 when the animal interacted with its social partner [Fig. 3e; W_(7)_ = − 16, p = 0.22, Wilcoxon matched-pairs signed-rank test, two-tailed]. Finally, a non-significant decrease was also observed in the spectral power of delta, theta, and beta band in the LFPs recorded in MD during pre- and post-LD [Fig. 3c, d; delta: W_(4)_ = − 10, p = 0.13, theta: W_(4)_ = − 10, p = 0.13, beta: W_(4)_ = − 8, p = 0.25, Wilcoxon matched-pairs signed-rank tests, two-tailed]. There were no statistically significant changes in the spectral power of LFPs recorded across all brain areas (AON, mPFC, NAc, MD, and CA1) when comparing pre-LF with post-LF phases (Fig. 3a, b).

### Differences in delta and theta band power during pre-LF, pre-LD, post-LF, and post-LD epochs

**Fig. 4:**
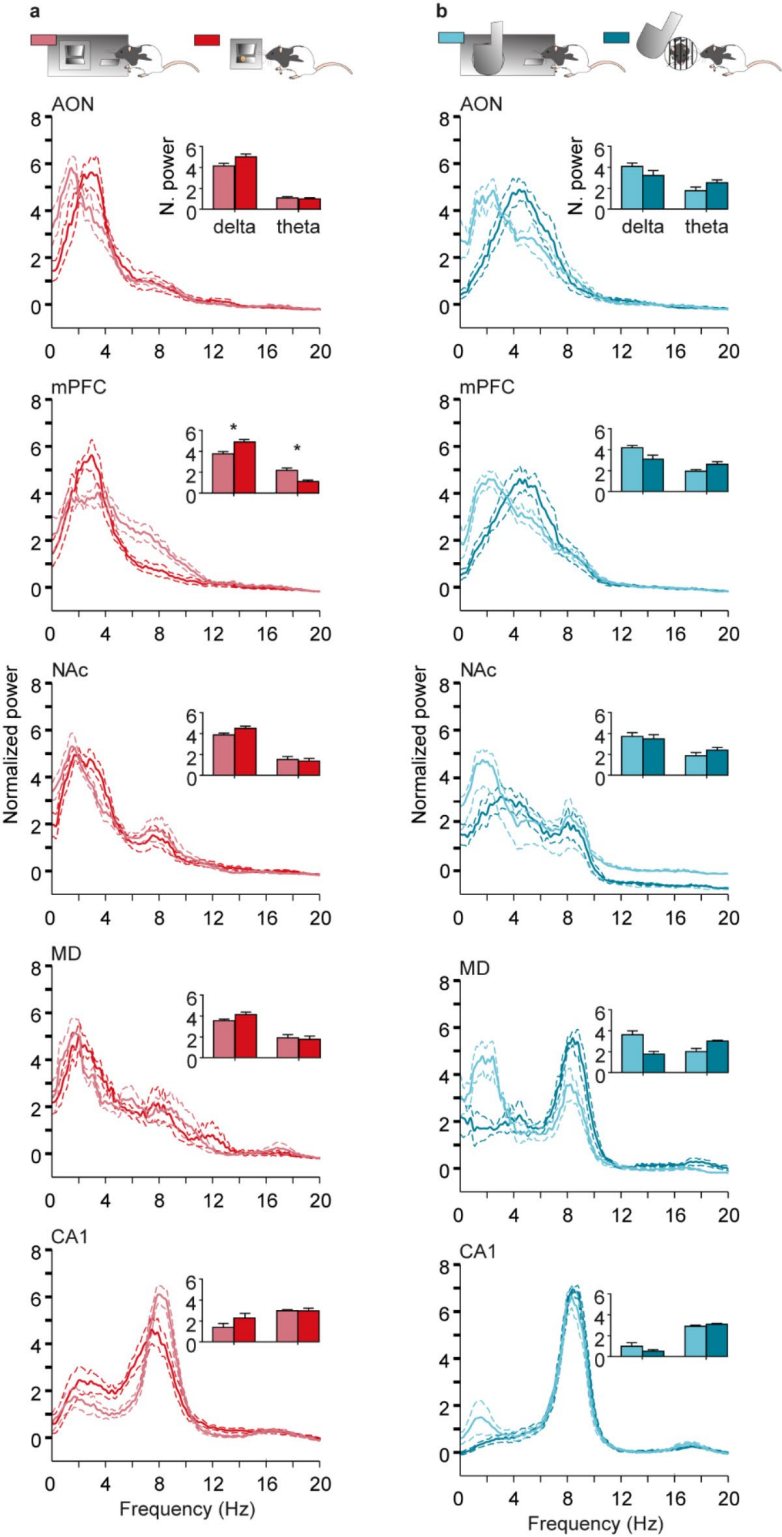
Power spectrum representation of LFPs recorded in the five selected brain areas. Inset figures represent the normalized mean power (Z-scored values) for delta (1.5–4 Hz) and theta (4–10 Hz) bands. (**a**) In light red Pre Lever-Feeder and in dark red Post Lever-Feeder. Significant differences between Pre Lever-Feeder and Post Lever-Feeder were as follows: in mPFC, delta band, *p* = 0.02, and theta band, *p* = 0.03. (**b**) Light blue Pre Lever-Door and dark blue Post Lever-Door. No significant differences were observed in delta and theta bands in any of the recorded brain areas. Continuous lines represent mean values, while the dashed lines represent ± s.e.m. values.

LFP spectral analysis performed during pre-LF and post-LF showed that there were significant differences in delta and theta bands only in mPFC [delta: F_(2.30, 11.48)_ = 7.70, p = 0.007, Greenhouse–Geisser correction (GGε) = 0.77, post hoc Tukey test, p = 0.03; theta: F_(1.99, 9.97)_ = 8.59, p = 0.007, GGε  = 0.66, post hoc Tukey test, p = 0.03], but that was not the case for pre-LD and post-LD (Fig. 4a,b). We did not find any other differences between pre- and post- task correspondent lever presses.

**Fig. 5:**
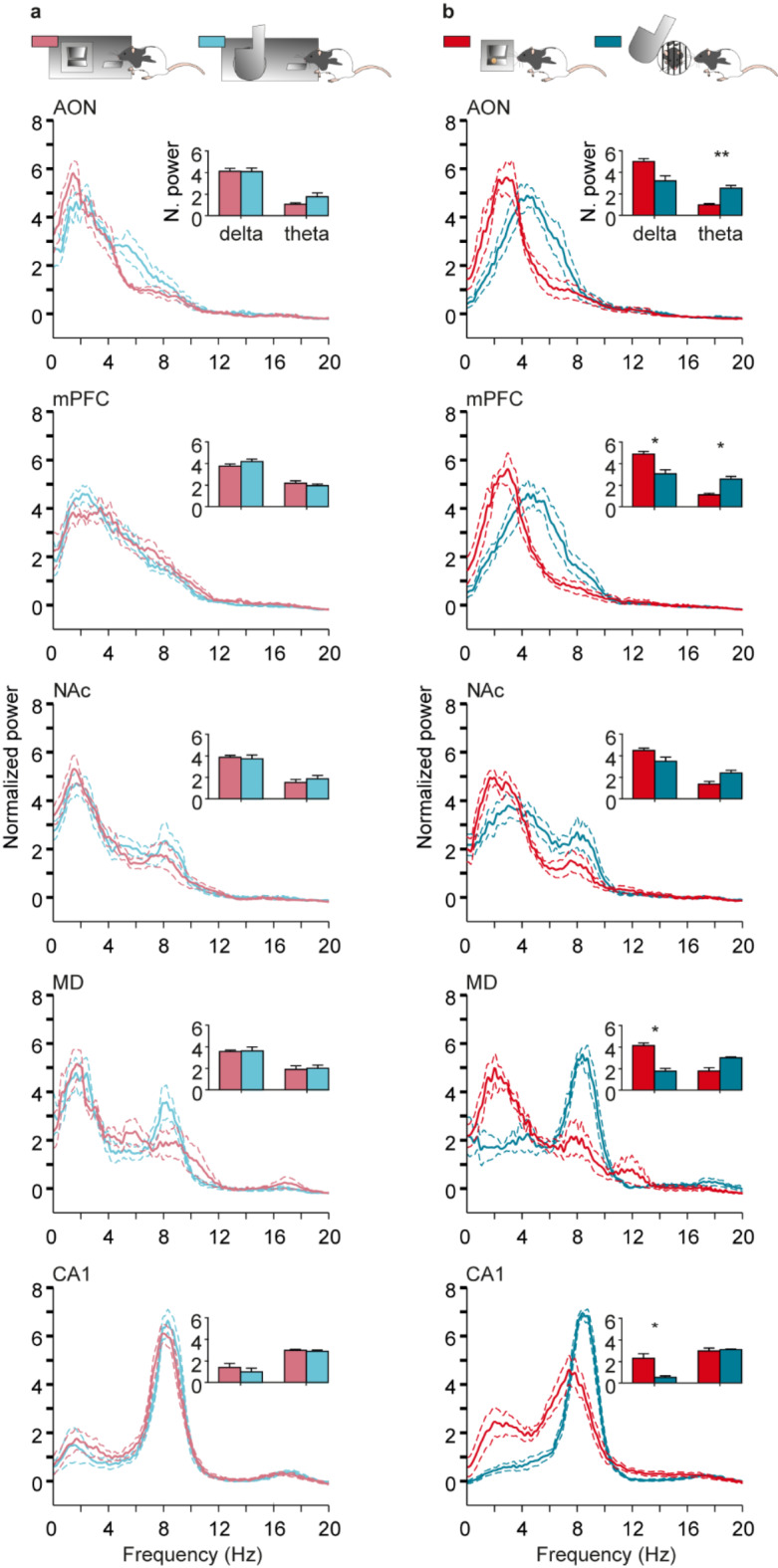
Power spectrum representation of LFPs recorded in the five selected areas for a comparison of values collected from Lever-Feeder vs Lever-Door responses. Inset figures represent normalized mean power (Z-scored values) for delta (1.5–4 Hz) and theta (4–10 Hz) bands. (**a**) In light red are shown Pre Lever-Feeder responses and in light blue Pre Lever-Door responses. No significant differences were observed in delta and theta bands in any brain area for these responses. (**b**) In dark red are represented Post Lever-Feeder responses and in dark blue are represented Post Lever-Door responses. Significant differences between Post Lever-Feeder and Post Lever-Door were as follows: in AON, theta band, *p* = 0.003; in mPFC, delta band, *p* = 0.01; theta band, *p* = 0.01; in MD, delta band, *p* = 0.03; and in CA1, delta band, *p* = 0.03. The continuous lines represent the mean, and the dashed lines represent ± s.e.m.

When comparing LFPs during the different lever-press conditions (Fig. 5), we did not observe differences between pre-LF and pre-LD, but, interestingly, we found differences after pressings for both levers in AON [theta: F_(1.57, 6.29)_ = 10.21, p = 0.01, GGε  = 0.52, post hoc Tukey test, p = 0.003], mPFC [delta: F_(2.30, 11.48)_ = 7.64, p = 0.007, GGε  = 0.77, post hoc Tukey test, p = 0.001; theta: F_(1.99, 9.97)_ = 8.59, p = 0.007, GGε  = 0.66, post hoc Tukey test, p = 0.02], MD [delta: F_(1.32, 3.97)_ = 13.94, p = 0.02, GGε  = 0.44, post hoc Tukey test, p = 0.03], and CA1 [delta: F_(2.15, 12.88)_ = 5.36, p = 0.02, GGε  = 0.72, post hoc Tukey test, p = 0.04] (Fig. 5b). In addition, there was a small shift in power peak frequency from pre-LD to post-LD in AON (pre-LD AON peak frequency: 2.4 Hz, post-LD AON peak frequency: 4.4 Hz) and mPFC (pre-LD mPFC peak frequency: 2.1 Hz, post-LD mPFC peak frequency: 4.4 Hz).

### Modulation by mPFC LFP phase of fast-gamma during food reward and slow-gamma during social interactions

**Fig. 6:**
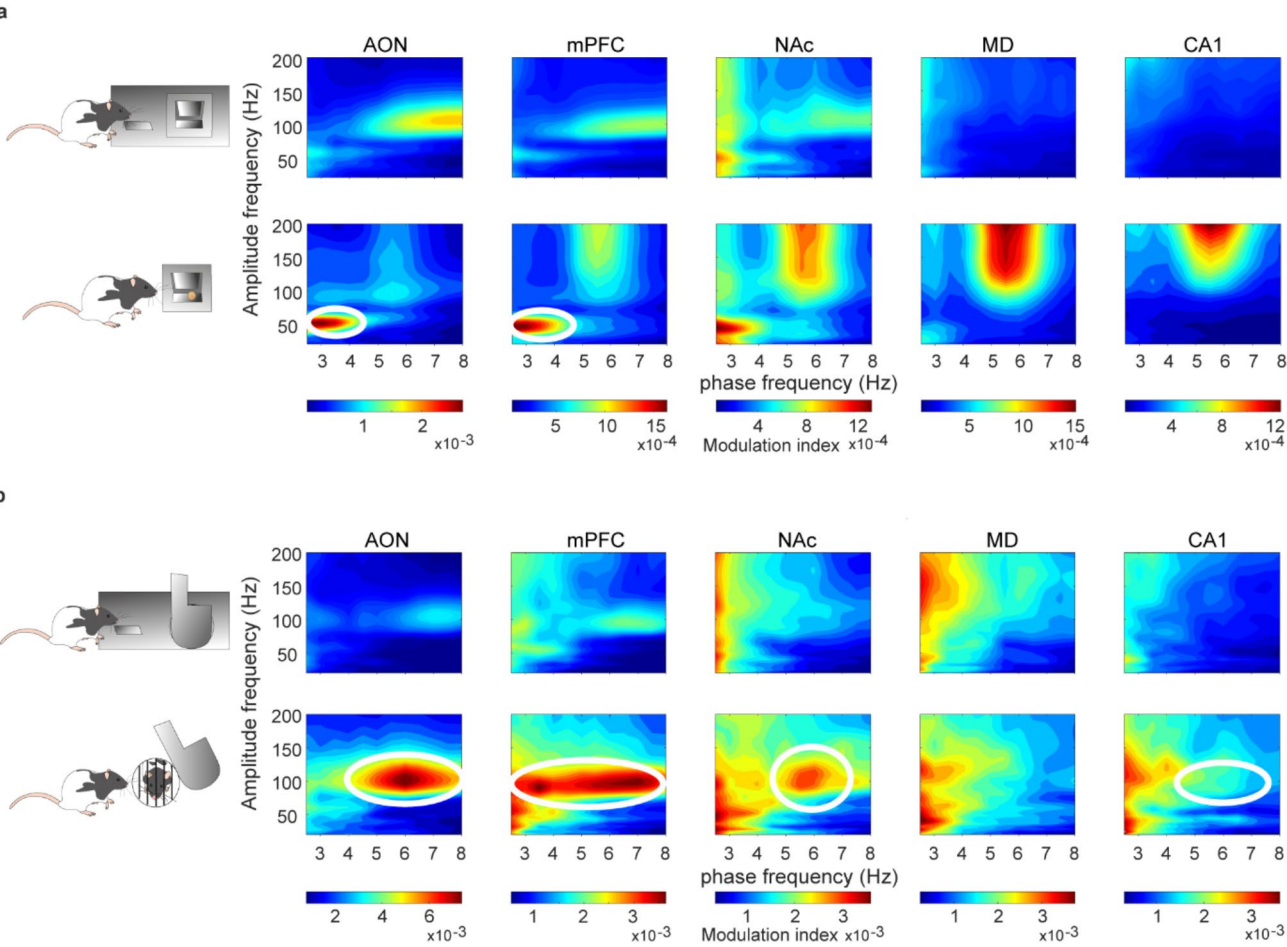
A representation of phase amplitude coupling across the different brain areas included in this study. Analyses were carried out from 3-s samples gathered from day 6 to day 10 from all animals (n = 10). (**a**) Pre and Post Lever-Feeder. Note that the phase of delta frequencies (2.5–4 Hz) of LFPs recorded in the mPFC modulate the amplitude of slow-gamma frequencies (30–60 Hz) of AON and mPFC during food rewards. (**b**) Pre and Post Lever-Door. For social interaction, theta frequencies (5 − 8 Hz) of mPFC modulated the amplitude of fast-gamma (80–110 Hz) of AON, mPFC, NAc and CA1 recordings. White circles surrounded the most significant changes in pre- and post-lever pressing.

**Fig. 7:**
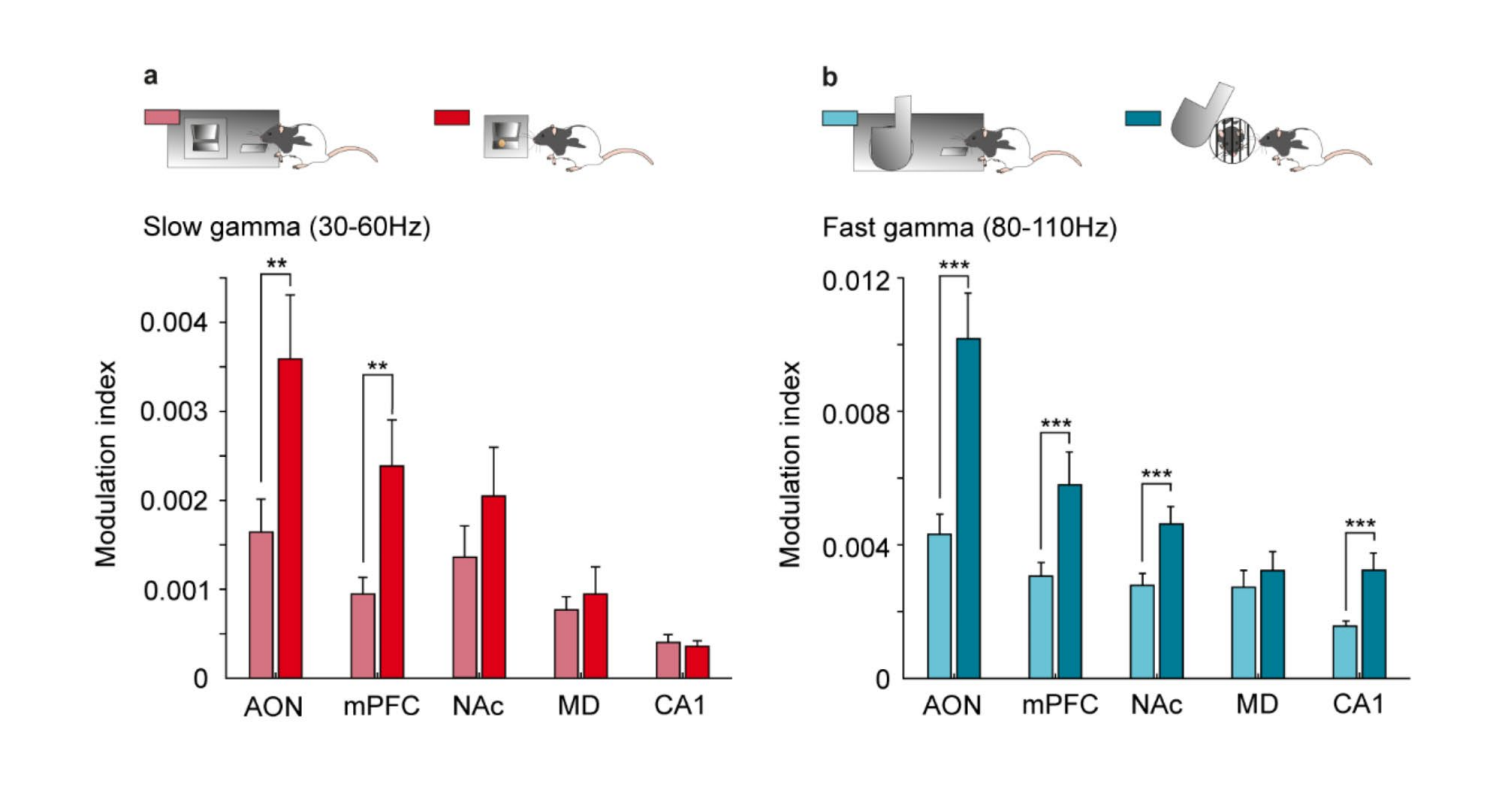
Quantification of the modulation index. (**a**) Pre (light red) and Post (dark red) Lever-Feeder during slow-gamma frequencies (30 − 60 Hz). Note the significant increase in AON (*p* = 0.006) and mPFC (*p* = 0.004). (**b**) Pre (light blue) and Post (dark blue) Lever-Door during fast-gamma frequencies (80–110 Hz). There was a significant increase in AON (*p* < 0.0001), mPFC (*p* = 0.0002), NAc (*p* < 0.0001), and CA1 (*p* < 0.0001) areas, after pressing the lever to access the social reward.

We investigated the cross-frequency coupling (phase-amplitude coupling) in LFP recordings collected from the mPFC with the other four recorded brain areas (AON, NAc, MD and CA1) (Fig. 6). A population analysis (Fig. 7) indicated that theta-to-gamma phase-amplitude coupling was higher in post-LF in AON [W_(21)_ = 153, *p* = 0.006, Wilcoxon matched-pairs signed-rank test, two-tailed] and mPFC [W_(21)_ = 161, *p* = 0.004, Wilcoxon matched-pairs signed-rank test, two-tailed] and also in post-LD in AON [W_(34)_ = 567, *p* < 0.0001, Wilcoxon matched-pairs signed-rank test, two-tailed], mPFC [W_(34)_ = 411, *p* = 0.0002, Wilcoxon matched-pairs signed-rank test, two-tailed], NAc [W_(34)_ = 439, *p* < 0.0001, Wilcoxon matched-pairs signed-rank test, two-tailed], and CA1 [W_(34)_ = 517, *p* < 0.0001, Wilcoxon matched-pairs signed-rank test, two-tailed] (Figs. 6 and 7). In addition, slow-gamma (30 − 60 Hz) frequencies were highly modulated by the mPFC LFP phase during food rewards, while fast-gamma frequencies (80 − 110 Hz) were most modulated during social interactions (Fig. 7).

## Discussion

Our study investigated the neural mechanisms underlying reward preferences in adult male rats by analyzing brain activity across multiple areas during lever-pressing tasks for food rewards versus social interactions. By examining the rats’ behavior and neural responses, we aimed to gain insights into the motivational and decision-making processes associated with each reward type. A consistent trend emerged where rats displayed a preference for food over social interaction, particularly evident during the final session of the experiment (Day 10). This preference aligns with recent findings in operant conditioning research conducted by Chow et al.^37^. Notably, we observed this preference in the absence of food restriction, with rats given ad libitum access to food and housed in isolation, suggesting a stronger motivational bias toward food rewards.

To minimize novelty effects, the experimental design maintained consistent conditions by using identical food pellets across trials and the same conspecific for all social interaction trials. This lack of variation in the social stimulus may have contributed to the gradual reduction in interest in the social interaction option over time.

To gain insights into the neural processes underlying these reward preferences, we recorded LFPs from selected brain sites well known for their involvement in associative learning and decision-making tasks. Specifically, we focused on the moments leading up to lever pressing and immediately afterwards—i.e., while obtaining the respective rewards. Using time–frequency analysis to examine all brain rhythms, we observed that NAc spectral power in the delta, theta, and beta bands diminished in the post-LD phase, when the door for social interaction opened and the experimental animal had the opportunity to interact with another animal. This finding contrasts with the results of Donnelly et al.^38^, where theta power in the NAc increased both during the waiting period and in relation to the response outcome. We hypothesize that this reduced spectral power may reflect a decreased interest in social interaction over time, as suggested by the rats’ performance across sessions.

In examining low-frequency rhythms in LFPs from the mPFC, we noted significant activity differences between the pre- and post-lever-press for food (pre-LF and post-LF) in both delta and theta bands. However, no statistical differences were observed between pre-LF and pre-LD (pre-lever-press for social interaction), which may suggest that the rats’ neural responses reflected a general expectation of reward, rather than a specific preference for either food or social interaction.

A novel contribution of our study is the differential spectral power observed across the five brain regions when comparing post-LF and post-LD conditions. This suggests that these regions process food and social rewards differently. Previously, we demonstrated that the mPFC plays a role in cooperative learning tasks in rats that incorporate both social interaction and food components^17^. In the present study, we identified differences in spectral characteristics of LFPs recorded from the AON, mPFC, mediodorsal thalamus (MD), and CA1. Notably, theta oscillations, peaking between 7 and 9 Hz, were observed in the CA1 region during social interactions, consistent with the oscillations associated with active locomotion and exploration, indicating an engaged response during rewarding social experiences^39,40^.

Given the known role of theta-range rhythms in facilitating inter-regional communication, we conducted cross-frequency coupling analysis to explore correlations between theta oscillations and gamma band activity in various brain areas. Focusing on interactions between the mPFC and other brain regions, we examined coupling between the phase of theta oscillations and the amplitude of gamma oscillations. A robust phase-amplitude coupling was observed during reward receipt, highlighting that the LFPs in these regions primarily reflected the processing of reward acquisition. Specifically, fast-gamma (80–110 Hz) and slow-gamma (30–60 Hz) oscillations appeared to play distinct roles in the acquisition of different rewards. These findings support previous research on theta–gamma modulation within the hippocampus related to contextual cues and reward characteristics^41–43^. In the same way, Donnelly et al.^38^, have reported an increase in the coupling of low-frequency oscillations (delta, theta) and gamma (50 − 60 Hz) oscillations in mPFC and NAc related to upcoming trial outcome and previous rewards in a 5-choice serial reaction time task. This modulation by the phase of the mPFC, a critical center for decision making^44^, highlights how various brain regions can be involved by this area in the encoding and processing information related to reward consummation. More specifically, we found that a greater number of areas were modulated by the mPFC during social interaction compared to food reward. Our findings contribute to the growing body of evidence supporting the role of theta–gamma modulation in reward-related processes across different brain regions^45,46^.

Finally, we acknowledge the limitations in using the number of rewards collected in an operant test as the sole measure of preference. For example, ten seconds of social interaction may not fully reflect its rewarding nature, while the lack of food restriction may have been insufficient to elicit a strong reward signal. Nonetheless, this simple task design allowed animals to respond with minimal researcher interference, supporting the reliability of their behavioral responses.

To deepen our understanding of reward preferences, and based on the outcome of our study, it would be interesting to investigate whether the same phase-amplitude coupling patterns are present in animals exhibiting a clear preference for either food or social interaction. Comparing the neural dynamics between these distinct preference groups could provide valuable insights into the underlying mechanisms and further elucidate the interplay between brain regions involved in reward processing.

## Methods

### Experimental animals

Experiments were carried out with male Lister Hooded rats (Charles River Laboratories, Barcelona, Spain). Rats were 3 months old (250 − 300 g) at the start of the experiments. They were housed individually, until the end of the experiment, with ad libitum access to food and water. They were under a 12:12 h light/dark cycle in standard temperature (21 ± 2 °C) and humidity (50 ± 10%) conditions.

### Ethics statement

All behavioral and electrophysiological experiments reported here were performed in accordance with the guidelines of the European Union Council (2010/63/EU) and following Spanish regulations (BOE 34/11,370–421, 2013) for the use of laboratory animals in chronic experiments. Experiments were also approved by the local Ethics Committee of Pablo de Olavide University and the Junta de Andalucía, Spain (codes 06/03/2018/025 and 06/04/2020/049). The present study was performed in accordance with ARRIVE guidelines (https://arriveguidelines.org). We hereby confirm that all experimental procedures were conducted following the above regulations and guidelines.

### Surgery

Following previously described surgical procedures^47^, rats were anesthetized with 1 − 2.5% isoflurane delivered via a rat anesthesia mask (David Kopf Instruments, Tujunga, CA, USA). Isoflurane was supplied from a calibrated Fluotec 5 vaporizer (Fluotec-Olmeda, Tewksbury, MA, USA), at a flow rate of 1 − 3 L/min of oxygen (AstraZeneca, Madrid, Spain). For LFP recordings, animals were held under stereotaxic guidance^48^ and chronically implanted with bipolar recording electrodes at the following brain sites: (i) anteroposterior (AP): 5.16 mm; lateral (L), 2.2 mm; and depth from brain surface (D) 4.4 mm for the AON; (ii) AP: 3.2 mm; L, 0.5 mm; and D, 3.8 mm for the mPFC; iii) AP: 1.7 mm; L, 1.3 mm; and D, 7.2 mm for the NAc; iv) AP: − 3.3 mm; L, 0.8 mm; and D, 5.2 mm for the MD; and v) AP: − 4.2 mm; L, 2.2 mm, and D 2.2 mm for the CA1. All electrodes were implanted in the right hemisphere (Fig. 1a, b). Electrodes were handmade from 50 µm, Teflon-coated, tungsten wire (Advent Research Materials, Eynsham, UK). Each electrode set consisted of two tungsten wires with a separation between tips of ~ 0.5 mm. Tips were stripped of the insulating Teflon coating for 50 µm to facilitate wire exposure and proper recordings. A silver wire was affixed to the bone with two stainless-steel screws to be used as ground during recording sessions. All the implanted wires were soldered to two six-pin sockets (RS Amidata, Madrid, Spain) that were fixed to the skull with six small stainless-steel screws and acrylic cement. After surgery, animals were returned to their cages for a 7-day recovery period before the beginning of the electrophysiology recordings. 

### Apparatus and recording procedures

Prior to any experimental test, animals were carefully handled for 3 min per day over at least 5 days. To measure rats’ preferences between food and social interaction without perturbations from experimenters, the experiments were carried out in two modified adjacent Skinner boxes (32 × 25.5 × 25.5 cm, l × w × h) (Fig. 1c). The Skinner boxes were separated by a guillotine door into two equal-size compartments. One of these Skinner boxes (Box 1) had two levers available: (i) a lever that, when pressed, provided access to food pellets, with a 1:1 fixed-ratio schedule (Lever-Feeder, LF); and ii) another lever (Lever-Door, LD) that, when pressed, allowed 10 s of visual and partial physical contact with an unfamiliar and non-cagemate rat located in the adjacent box (Box 2) (Fig. 1d). This was achieved by the mechanical opening of the guillotine door which gave access to a stainless-steel grid separating the two boxes. When one of the two levers was pressed, the two of them retracted and remained hidden for 15 s. Thus, if, for example, LD was pressed, the door was opened for 10 s, and after 5 additional seconds, the two levers came out and were again available to be pressed. We had two additional animals used as social stimulus that were interchanged between sessions each day. These animals were placed in Box 2 and had no implanted electrodes.

Every testing session had a duration of 20 min for 10 consecutive days (Fig. 1e). All operant sessions were monitored and recorded using the MED-PC program (MED Associates, St. Albans, VT, USA) and a video capture system synchronized with the recording system (Sony HDR-SR12E, Tokyo, Japan). Locomotor activity was analyzed offline using Tracker Video Analysis and Modeling Tool^49^, and heatmap representation was generated using MATLAB. A dim light was used to reduce stress of the experimental animals^50^. After each testing session, all apparatus was cleaned with soapy water and with 70% alcohol solution to prevent possible biasing effects of odor left by previous subjects^51^.

Implanted animals received two habituation sessions (the first without and the second with wires connecting the implanted electrodes to the recording system) before the first experimental session. The aim was to habituate the animals to the Skinner box, to the recording system, and to the experimental situation.

Experimental animals presenting aggressive behaviors and/or forcing the opening of the door to interact directly with the accompanying rat were removed from the study. Experimental animals always ate the food pellets that appeared after pressing the Lever-Feeder.

### Histology

At the end of the programmed experiments, rats were deeply anesthetized with a mixture of ketamine (100 mg/kg) and medetomidine (0.1 mg/kg) and perfused transcardially with saline and 4% paraformaldehyde in phosphate-buffered saline (PBS, 0.1 M, pH 7.4). Brains were removed and cryoprotected with 30% sucrose in PB for a few days until sectioning. Coronal Sects. (50 µm) were cut with a sliding freezing microtome (Leica SM2000R, Nussloch, Germany). Selected sections that included the implanted areas were mounted on gelatinized glass slides and stained afterwards using Nissl technique with 0.1% toluidine blue to reveal the final location of recording electrodes in all targeted brain areas (Fig. 1b).

### Data collection and analysis

The criterion established was observing the animals go directly to collect the corresponding reward after pressing a lever. If an animal did not collect the pellet of food, or did not turn to the guillotine door, we excluded it from the analysis; rats presenting problems in the recording system were also excluded from the experiment. A total of 10 rats reached the selected criterion by the 6th session on and were used for the analytical procedures (Fig. 2). LFP activities were recorded using Grass P511 differential amplifiers with a bandwidth of 0.1 Hz–3 kHz (Grass-Telefactor, West Warwick, RI, USA). LFPs and one-volt rectangular pulses corresponding to lever presses, door opening, pellet delivery, and video recordings, were stored digitally on a computer through analog-to-digital converters (CED 1201 Plus; Cambridge Electronics Design). LFPs were sampled at 5 kHz.

Data were analyzed off-line with the Spike2 software (Cambridge Electronics Design). The samples of LFP selected and analyzed all had a duration of 3 s and were collected before pressing Lever-Feeder (pre-LF), before pressing Lever-Door (pre-LF), after pressing Lever-Feeder and obtaining the food pellet (post-LF), and after pressing Lever-Door and opening the door for social interaction (post-LD) (Fig. 1f). Epochs were selected automatically using custom MATLAB script that tracked TTL pulses synchronized with lever presses (Fig. 1f). These selections were then manually reviewed to confirm the corresponding reward consumption. Before computing the power spectrum, data were normalized (Z-scored). The frequency bands selected for this study were: delta (1.5–4 Hz), theta (4–10 Hz), beta (10 − 30 Hz), slow-gamma (30 − 60 Hz), and fast-gamma (80 − 110 Hz). These frequency ranges were selected based on prior research ^52^, with gamma divided into sub-bands to explore their potential roles in distinct neural processes and associated behaviors, as previously described^53–55^.

To compute time–frequency representations (spectrograms; *mtspecgramc* function; Fig. 3) and the frequency domain characteristics (power spectrum; *mtspectrumc* function; Figs. 4 and 5) for each brain region and from the four conditions studied, we used the multi-taper method of the Fourier transform from Chronux toolbox^56^ software (version 2.12, Website: http://chronux.org/) in the MATLAB program. The power spectrum and spectrograms were computed using 5 tapers (K), and time-bandwidth (TW) of 3 (params.tapers = [TW = 3 K = 5]). A moving window of 0.5 s shifted in 10 ms increments (movingwin = [0.5 0.01]) was also necessary for spectrogram analyses. With *randsample* from MATLAB, the same number of samples for both food and social interaction (during pre- and post-) for each animal each session day (from day 6 on, inclusively) were used in the spectrogram (Fig. 3) and power spectrum analyses (Figs. 4 and 5). AON (n = 60 samples; 5 rats); mPFC (n = 57 samples; 6 rats); NAc (n = 73 samples; 7 rats); MD (n = 37 samples; 4 rats); and CA1 (n = 80 samples; 7 rats). Phase-amplitude coupling (PAC) (Fig. 6) was quantified by a modulation index (MI). MI is a measure used to quantify the amplitude modulation of high-frequency activity within the phase of low-frequency oscillations. Additional details of these analytical procedures have been published by some of us elsewhere^21,47,57,58^.

Collected results were processed for statistical analysis using Prism 6 (GraphPad Software, Inc, La Joya, CA, USA). For statistical comparisons, a two-way repeated-measures ANOVA with time (Days) and lever type (Lever-Feeder and Lever-Door) as within-subjects factors was conducted to examine main and interaction effects on lever press counts, with Tukey’s post hoc test applied. For each brain area, a repeated-measures one-way ANOVA with treatment (pre-LF, post-LF, pre-LD, and post-LD) as a within-subjects factor was used to assess effects on neural activity levels, with individual differences accounted for as a random factor and Tukey’s post hoc test for multiple comparisons. A two-tailed Wilcoxon matched-pairs signed-rank test was used for non-normally distributed data, as determined by the Shapiro–Wilk test. For all the statistical tests, the significance level (p value) is indicated. *p < 0.05; **p < 0.01; ***p < 0.001. Unless otherwise indicated, data are always represented as the mean ± s.e.m.

## Electronic supplementary material

Below is the link to the electronic supplementary material.

Supplementary Material 1

Supplementary Material 2

## Data Availability

Derived data in support of the findings of this study, text mining data, and word embedding are available from the corresponding author upon reasonable request.
