# Supplementary material for: Cortical and subcortical activities during food rewards versus social interaction in rats
Source: Sci Rep. 2025 Feb 5;15:4389. doi: 10.1038/s41598-025-87880-1 (PMC11799384; doi:10.1038/s41598-025-87880-1)
Supplement: Supplementary file 1 — Supplementary Material 1 [file 41598_2025_87880_MOESM1_ESM.docx]

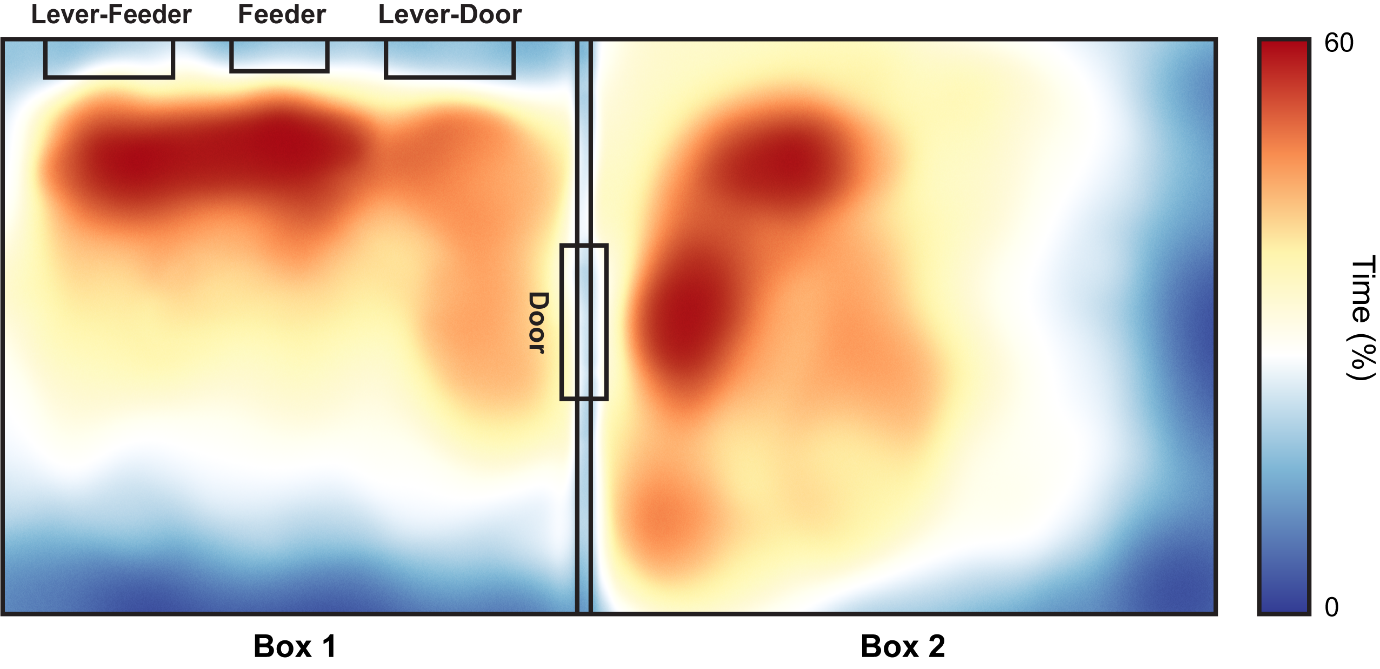


Supplementary Figure S1. Heatmap showing the time spent by the experimental animal in Box 1 and the social stimulus rat in Box 2 during a single session. The heatmap was generated in MATLAB using data analyzed offline with the Tracker Video Analysis and Modeling Tool.
