# Supplementary figures and images for: Cortical and subcortical activities during food rewards versus social interaction in rats
Source: Sci Rep. 2025 Feb 5;15:4389. doi: 10.1038/s41598-025-87880-1 (PMC11799384; doi:10.1038/s41598-025-87880-1)

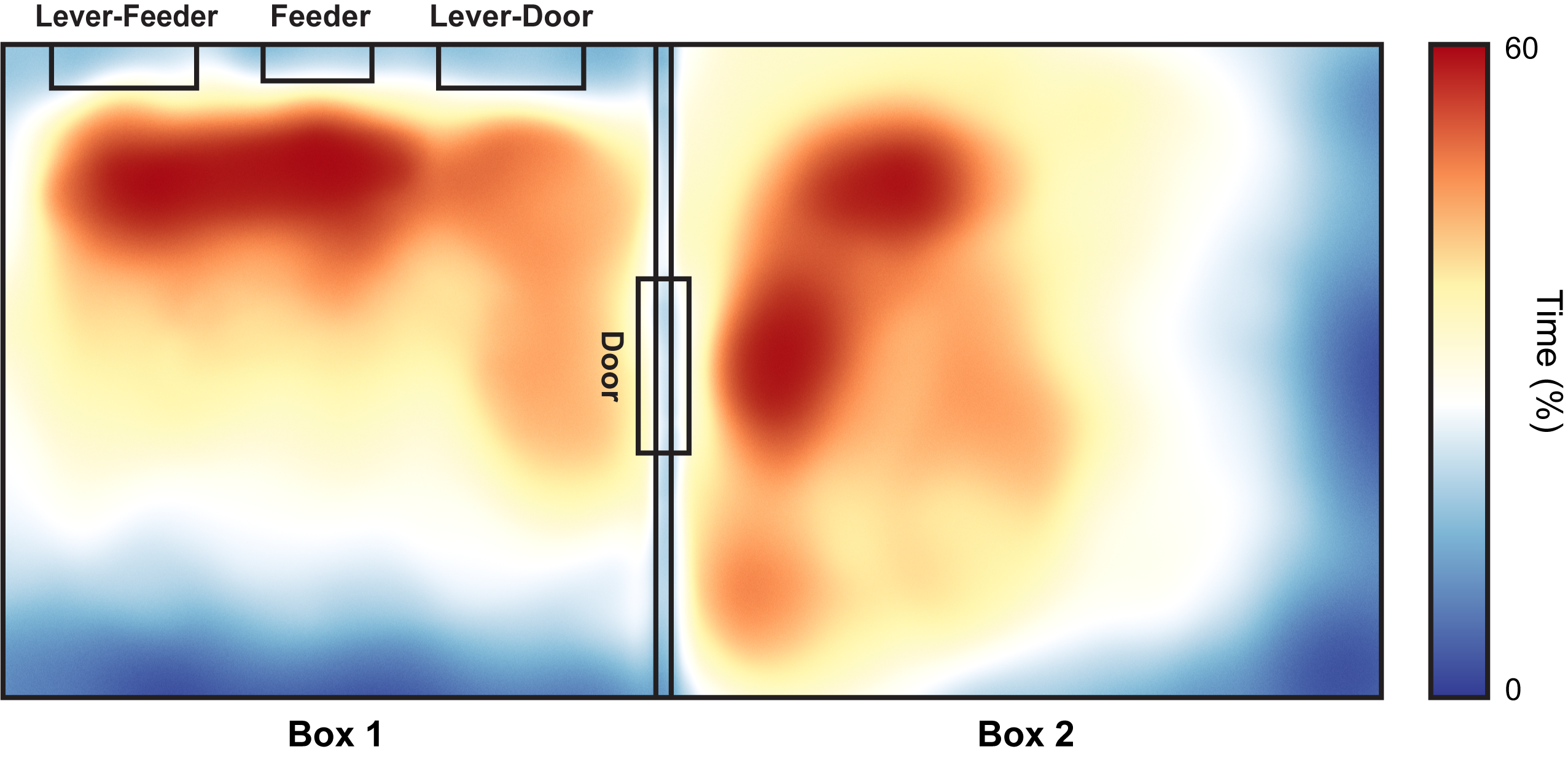

Supplement: Supplementary file 2 — Supplementary Material 2 [file 41598_2025_87880_MOESM2_ESM.tif]
